# Core High-Risk Foot Profiles and Surgery-Coded Care-Intensity Indicators Among Hajj Pilgrims Presenting with Foot and Ankle Conditions: A Presentation-Level Analysis

**DOI:** 10.3390/healthcare14121782

**Published:** 2026-06-20

**Authors:** Mohammed F. AlGabgab, Naif Alqurashi, Majed Alqahtani, Moharmis M. Alolyani, Osama A. Samarkandi

**Affiliations:** 1 Department of Basic Sciences, Prince Sultan bin Abdulaziz College for Emergency Medical Services, King Saud University, Riyadh 11466, Saudi Arabia; malgabgab@ksu.edu.sa (M.F.A.); maalqahtani@ksu.edu.sa (M.A.); 2 Department of Accidents and Trauma, Prince Sultan bin Abdulaziz College for Emergency Medical Services, King Saud University, Riyadh 11466, Saudi Arabia; nalqurashi1@ksu.edu.sa; 3 Department of Emergency Medical Services, Prince Sultan bin Abdulaziz College for Emergency Medical Services, King Saud University, Riyadh 11466, Saudi Arabia; malolyani@ksu.edu.sa

**Keywords:** Hajj, mass gatherings, diabetic foot, foot ulcer, neuropathy, foot and ankle conditions, referral, triage, wound care, healthcare preparedness

## Abstract

Background/Objectives: Foot and ankle presentations during Hajj occur in a dense mass-gathering environment where prolonged walking, heat exposure, crowding, variable footwear, and limited self-care can interact with chronic disease and wound vulnerability. Previous Hajj studies have described foot injuries and diabetes-related complications, but less is known about whether simple high-risk foot documentation flags identify presentation records with higher care-pathway intensity. The primary objective was to estimate the presentation-level burden of core high-risk foot profiles among pilgrims presenting with foot and ankle conditions during Hajj 2025. Secondary objectives were to evaluate associations with a surgery-coded care-intensity indicator, hospital referral, and component heterogeneity. Methods: This observational presentation-level analysis included 3957 foot and ankle presentation records. The unit of analysis was the presentation/case record, not a unique individual pilgrim. A core high-risk foot profile was defined as diabetes, neuropathy, diabetic foot ulcer, foot ulcer, complications of open wound, or osteomyelitis. The primary outcome was a surgery-coded care-intensity indicator, defined solely from treatment documentation containing “Surgery” and interpreted as a care-pathway proxy rather than confirmed operating-room surgery. Logistic regression estimated crude and adjusted odds ratios (ORs); exploratory risk-category analyses assessed heterogeneity within the composite profile. Results: Core high-risk foot profiles were identified in 1793/3957 presentations (45.3%). The primary outcome occurred in 239/1793 high-risk presentations (13.3%) and 201/2164 non-high-risk presentations (9.3%), an absolute difference of 4.0 percentage points. The crude OR was 1.50 (95% CI 1.23–1.83; *p* < 0.001). The association persisted in the primary adjusted model (adjusted OR 1.47; 95% CI 1.20–1.79; *p* < 0.001) and in the extended clinical sensitivity model (adjusted OR 1.47; 95% CI 1.20–1.80; *p* < 0.001). Care pathways and secondary outcomes are summarized was also more frequent in high-risk presentations (12.2% vs. 9.8%; crude OR 1.28; 95% CI 1.05–1.57; *p* = 0.017). Exploratory category analysis showed that chronic-risk-only presentations had a primary outcome rate similar to non-high-risk presentations (9.0% vs. 9.3%), whereas ulcer/wound/deep-infection presentations had a higher rate (17.3%; crude OR 2.04; 95% CI 1.63–2.55; *p* < 0.001). Model discrimination was modest (C-statistics 0.55–0.64). Conclusions: Core high-risk foot flags were common among Hajj foot and ankle presentation records and were associated with surgery-coded care-intensity and referral documentation. However, the composite was clinically heterogeneous, the outcome was not a validated surgery endpoint, and the models were not prediction tools. These findings support cautious use of high-risk foot flags as operational prompts for assessment and pathway planning rather than as standalone clinical risk estimates.

## 1. Introduction

Hajj is one of the world’s largest recurring religious mass gatherings. The 1446H/2025 Hajj season included 1,673,230 pilgrims, of whom approximately 90% arrived from outside Saudi Arabia, creating a dense, multinational, and time-bounded health-service environment [1]. Mass gatherings can strain host health-system capacity and require planning for infectious, environmental, traumatic, and non-communicable disease risks [2,3].

Foot and ankle conditions are particularly relevant during pilgrimage because rituals commonly involve prolonged walking, standing, crowd movement, heat exposure, and footwear-related stress. Prior Hajj and pilgrimage studies have described foot injuries, blisters, ulcers, musculoskeletal complaints, and diabetes-related complications [4,5,6,7,8,9]. However, many prior reports are primarily descriptive and do not clearly distinguish general foot and ankle presentations from presentation records that contain high-risk chronic disease, neuropathic, ulcer/wound, or deep-infection features requiring different triage and escalation logic.

The clinical rationale for a high-risk foot profile is based on diabetes-related foot disease principles: neuropathy, ulceration, infection, peripheral arterial disease, wound depth, and prior tissue breakdown can influence the urgency of assessment, offloading, wound care, and referral [10,11,12,13,14,15,16,17]. In Hajj, these risks may be amplified by intense mobility demands, heat, crowding, and variable capacity for self-inspection and follow-up among multinational pilgrims [6,18,19]. Therefore, a diabetic-foot-only frame may be too narrow for first-contact Hajj triage, but an overly broad composite may introduce clinical heterogeneity if not interpreted cautiously.

The primary research question was as follows: among pilgrims presenting with foot and ankle conditions during Hajj, what proportion of presentation records meet a core high-risk foot profile flag definition, and is this flag associated with a surgery-coded care-intensity indicator? Secondary questions were whether the profile was associated with hospital referral and other care-pathway indicators, and whether exploratory component-based categories suggested heterogeneity within the composite profile. Because the treatment field did not verify definitive surgery, the primary outcome was defined and interpreted as a surgery-coded care-intensity proxy, not a confirmed operative event or an independently validated escalation endpoint.

## 2. Materials and Methods

### 2.1. Study Design and Setting

This was an observational presentation-level analysis of foot and ankle presentation records collected during Hajj 2025. The analysis followed STROBE reporting principles for observational studies [20,21]. Records were derived from field-collector records and health information system sources and included presentations from Makkah and the holy sites, including Mina, Arafat, and Muzdalifah.

### 2.2. Participants and Unit of Analysis

The analytic dataset included 3957 presentation records. The unit of analysis was the presentation/case record, not the individual pilgrim and not the total Hajj pilgrim population. Accordingly, all percentages are presentation-level proportions among foot and ankle presentations, not population prevalence estimates among all pilgrims. The dataset did not contain a verified unique patient identifier suitable for longitudinal linkage; therefore, repeated presentations by the same pilgrim could not be identified or clustered. This violates the assumption that every record necessarily represents an independent person and was considered when interpreting standard errors, confidence intervals, *p*-values, and generalizability.

### 2.3. Data Processing, Quality Control, and Missing Data

Source variables were harmonized into a row-level analysis file. Location names, previous-pilgrimage categories, injury types, footwear condition, radiographic results, diagnoses, treatment fields, and follow-up fields were standardized before deriving binary analytic flags. Text and category fields were trimmed, standardized, and mapped using prespecified rule-based logic. The surgery-coded care-intensity indicator, referral, casting, follow-up, fracture, diabetes, neuropathy, ulcer/wound, osteomyelitis, pressure-ulcer, and infection flags were derived from specified source fields and reconciled against aggregate row counts in the analysis workbook and supplementary dictionary. Range and logic checks were applied to age, BMI, pain score, binary flags, denominators, and mutually incompatible response patterns. A formal independent clinical adjudication sample was not available; therefore, text-derived exposure and outcome flags remain vulnerable to misclassification. The primary regression model used all 3957 records because age, sex, BMI, presentation site, exposure, and primary outcome were complete in the analysis file. Blank pre-existing-condition fields were interpreted as not documented for the purpose of binary flag derivation, which may underestimate diabetes or neuropathy and is treated as a potential documentation bias.

### 2.4. Exposure Definition

The primary exposure was a core high-risk foot profile flag, defined a priori as the presence of at least one of the following: pre-existing diabetes, pre-existing neuropathy, diabetic foot ulcer diagnosis, foot ulcer diagnosis, complications of open wound, or osteomyelitis. These components were selected because they reflect chronic metabolic risk, neuropathic vulnerability, ulcer/wound tissue breakdown, or potential deep infection. However, the components are not clinically equivalent. The profile was therefore treated as a broad first-contact documentation flag for service planning, not as a validated severity score, a weighted risk instrument, or a single homogeneous clinical syndrome.

To address component heterogeneity, an exploratory post hoc risk-category analysis separated presentation records into non-high-risk, chronic-risk-only (diabetes and/or neuropathy without ulcer, open-wound complication, or osteomyelitis), and ulcer/wound/deep-infection categories. This analysis was descriptive and hypothesis-generating and was not used to redefine the primary exposure.

### 2.5. Outcome Definitions

The primary outcome was a surgery-coded care-intensity indicator, operationalized as the treatment field containing “Surgery”. Because the source field does not distinguish operating-room surgery from debridement, surgical assessment, procedural care, or referral-related surgical coding, this variable was interpreted as a care-pathway proxy rather than definitive proof of operative surgery or clinically verified escalation of care. The term “surgery-coded” is retained to preserve the source-field definition, while the interpretation is deliberately neutral.

Secondary outcomes were referral to hospital, a clinical/care-intensity proxy, planned follow-up, and confirmed fracture. The clinical/care-intensity proxy was defined as confirmed fracture, observed deformity, casting, referral, or surgery-coded care-intensity indicator. This composite was used only as a supportive care-intensity measure and was not interpreted as an independently validated severity scale.

### 2.6. Covariates

Covariates included age, sex, BMI, presentation site, pain score, footwear problem, swelling, bruising, deformity, and confirmed fracture. Presentation site was modelled as Makkah versus non-Makkah in the primary adjusted model because one raw-site category had zero primary outcome events, making raw-site estimates unstable. To improve transparency, site-specific event counts and sensitivity models using alternative site handling were added. Variables such as diabetes duration, glycemic control, vascular status, wound depth, infection grade, peripheral arterial disease, baseline functional status, microbiology, and time-to-care were not available in the source dataset and could not be included. Accordingly, adjusted estimates were interpreted as descriptive care-pathway associations rather than independent clinical risk estimates.

### 2.7. Statistical Analysis

Categorical variables are summarized as frequencies and percentages, and continuous variables as mean (standard deviation) or median (interquartile range), as appropriate. Group comparisons are descriptive and used chi-square, Fisher exact, Welch *t*-test, or Mann–Whitney U tests according to variable structure. Crude odds ratios were calculated for binary outcomes. Logistic regression estimated crude, primary adjusted, and extended clinical sensitivity associations between the high-risk profile and the primary outcome. The primary adjusted model included high-risk profile, age, sex, BMI, and Makkah versus non-Makkah presentation site. The extended clinical sensitivity model additionally included pain score, footwear problem, swelling, bruising, deformity, and confirmed fracture.

C-statistics were reported to describe discrimination but were not used to present the models as prediction tools. Sensitivity, specificity, predictive values, calibration, and validation statistics were not presented because the study did not develop or validate an individual-level prediction model. Site-specific primary outcome counts were tabulated. As sensitivity analyses for the site variable, the association between the core high-risk flag and the primary outcome was re-estimated without site adjustment and after excluding Arafat while modelling Makkah, Mina, and Muzdalifah as categorical site covariates. Two-sided *p*-values < 0.05 were considered statistically significant. Statistical analyses were conducted using Python (version 3.13.5; Python Software Foundation, Wilmington, DE, USA), and tabular quality checks were summarized in Microsoft Excel (Microsoft Corporation, Redmond, WA, USA). Exploratory component and risk-category analyses were interpreted cautiously because components were not mutually exclusive and the dataset lacked detailed severity markers.

### 2.8. Ethics

The study was conducted in accordance with the Declaration of Helsinki and approved by the Riyadh Second Health Cluster Institutional Review Board (IRB Log Number: 25-259E; approval date: 12 May 2025). Consent procedures were conducted in accordance with the IRB-approved protocol. The analysis used de-identified presentation-level data, and no personal identifying information is reported in the manuscript, tables, or figures.

## 3. Results

### 3.1. Study Population and High-Risk Profile Burden

Among 3957 foot and ankle presentation records, 1793 (45.3%) met the core high-risk foot profile definition and 2164 (54.7%) did not. This 45.3% estimate is a presentation-level proportion among foot and ankle records, not a prevalence estimate among all Hajj pilgrims. Baseline characteristics are summarized in Table 1, and components of the core high-risk foot profile are shown in Table 2. The most frequent components were pre-existing diabetes (1040; 26.3%), diabetic foot ulcer diagnosis (381; 9.6%), pre-existing neuropathy (353; 8.9%), and foot ulcer diagnosis (264; 6.7%).

### 3.2. Care Pathways and Primary Outcome

The surgery-coded care-intensity indicator occurred in 440/3957 presentations (11.1%). It was more common among high-risk presentations than non-high-risk presentations (13.3% vs. 9.3%), corresponding to an absolute difference of 4.0 percentage points and a crude OR of 1.50 (95% CI 1.23–1.83; *p* < 0.001). Care pathways and secondary outcomes are summarized in Table 3. Referral to hospital was also more frequent in the high-risk group (12.2% vs. 9.8%; absolute difference 2.4 percentage points; crude OR 1.28; 95% CI 1.05–1.57; *p* = 0.017).

### 3.3. Regression Analysis

**Figure 1:**
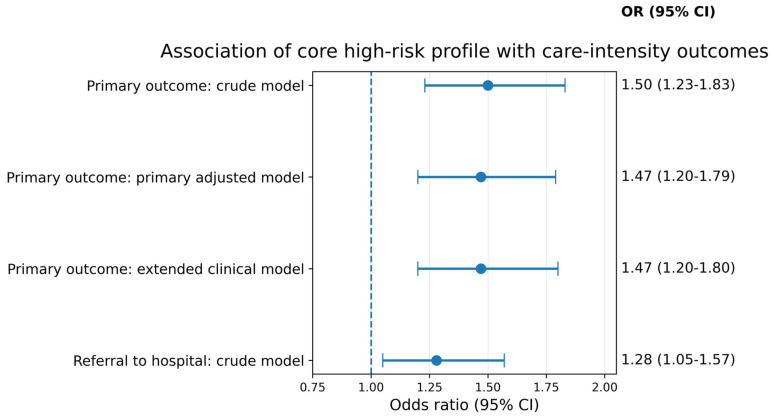
Forest plot of the association between the core high-risk foot profile and care-intensity outcomes. Odds ratios compare high-risk versus non-high-risk presentation records. The primary outcome is the surgery-coded care-intensity indicator.

In the crude model, the high-risk profile flag was associated with higher odds of the surgery-coded care-intensity indicator (OR 1.50; 95% CI 1.23–1.83; *p* < 0.001). This association remained statistically significant after adjustment for age, sex, BMI, and Makkah versus non-Makkah site (adjusted OR 1.47; 95% CI 1.20–1.79; *p* < 0.001). The extended clinical sensitivity model yielded a similar estimate (adjusted OR 1.47; 95% CI 1.20–1.80; *p* < 0.001). Figure 1 and Table 4 summarize these regression estimates. However, model discrimination was modest, with C-statistics ranging from 0.55 to 0.64; therefore, these models should be interpreted as explanatory association models, not as standalone prediction tools or individual-level triage algorithms.

### 3.4. Sensitivity and Exploratory Component Analyses

Primary outcome events differed by presentation site: Makkah 401/3058 (13.1%), Mina 20/371 (5.4%), Arafat 0/400 (0.0%), and Muzdalifah 19/128 (14.8%). Because Arafat had no primary outcome events, raw-site modelling is susceptible to separation and unstable site coefficients. Sensitivity analyses showed that the high-risk association was similar without site adjustment (adjusted OR 1.47; 95% CI 1.21–1.80; *p* < 0.001) and after excluding Arafat and modelling Makkah, Mina, and Muzdalifah as categorical site covariates (adjusted OR 1.47; 95% CI 1.20–1.80; *p* < 0.001). An expanded high-risk definition that added infection-type injury and early pressure-ulcer diagnosis classified 2330/3957 presentations (58.9%) as high-risk, but the association with the primary outcome was weaker and not statistically significant (crude OR 1.14; 95% CI 0.93–1.39; *p* = 0.241). In exploratory component-category analysis, chronic-risk-only presentations had a primary outcome rate similar to non-high-risk presentations (9.0% vs. 9.3%; crude OR 0.97; 95% CI 0.73–1.27; *p* = 0.834), whereas ulcer/wound/deep-infection presentations had a higher rate (17.3%; crude OR 2.04; 95% CI 1.63–2.55; *p* < 0.001). These exploratory findings suggest clinical heterogeneity within the composite profile and are reported in Supplementary File.

## 4. Discussion

### 4.1. Principal Findings

This analysis clarifies three main findings. First, 45.3% of foot and ankle presentation records during Hajj met the core high-risk foot profile flag definition; this is a presentation-level burden among patients already presenting with foot and ankle conditions, not prevalence among all pilgrims. Second, the high-risk flag was associated with higher odds of a surgery-coded care-intensity indicator and hospital referral, although the absolute differences were moderate. Third, the composite profile was clinically heterogeneous: chronic-risk-only records had a primary outcome rate similar to non-high-risk records, whereas ulcer/wound/deep-infection records had a higher rate. These findings support cautious service-planning interpretation rather than individual-level prediction.

### 4.2. Interpretation of the High-Risk Profile

A high-risk profile approach may be useful in Hajj because first-contact clinicians and field teams often need simple prompts that can be recognized quickly from presenting diagnoses and documented chronic conditions. Diabetes, neuropathy, ulceration, open-wound complications, and osteomyelitis are clinically coherent as broad high-risk foot markers because they align with established diabetes-related foot disease concepts of tissue breakdown, loss of protective sensation, infection, vascular compromise, and escalation need [10,11,12,13,14,15,16,17]. However, the composite is heterogeneous and should not be treated as a single clinical entity. Uncomplicated diabetes is not clinically equivalent to active ulceration, open-wound complications, or osteomyelitis, and the exploratory category analysis supports this caution: ulcer/wound/deep-infection presentations showed higher care-intensity rates than chronic-risk-only presentations. Therefore, the profile should not be interpreted as a validated severity scale, weighted risk score, or independent clinical risk estimator.

### 4.3. Interpretation of the Primary Outcome

The primary outcome was intentionally reframed as a surgery-coded care-intensity indicator. The data source identifies records in which the treatment field contains “Surgery”, but it does not verify whether the event was definitive operating-room surgery, bedside debridement, surgical assessment, procedural care, or referral-related surgical coding. This uncertainty limits clinical interpretation of the exact intervention type. The finding should therefore be understood as evidence of increased care-pathway intensity or escalation coding among high-risk presentation records, not as a confirmed surgery rate or clinically adjudicated endpoint.

### 4.4. Operational Implications for Hajj Health Services

The operational value of the findings lies in early recognition and pathway design, not in replacing clinical judgement. High-risk foot flags may support assessment prompts for diabetes/neuropathy history, focused foot inspection, ulcer/wound assessment, infection red flags, offloading needs, and early referral criteria. Preventive and care-pathway methods that may reduce burden include pre-Hajj and point-of-care education about daily foot inspection, avoiding barefoot walking, appropriate footwear, early reporting of blisters and wounds, protection of pre-ulcerative lesions, glucose-aware clinical assessment, and rapid escalation for ulceration, open-wound complications, suspected infection, deformity, fracture, or osteomyelitis [11,12,18,19]. These implications should be considered as hypotheses for service design rather than as interventions directly tested by the present study.

### 4.5. Comparison with the Previous Literature

Prior studies have described Hajj-related foot injuries, diabetes complications, and musculoskeletal trauma patterns among pilgrims [4,5,6,7,8,9]. The present study extends this literature by focusing on a presentation-level operational profile linked to care-intensity and referral outcomes rather than only describing injury types. The revised framing is also consistent with contemporary diabetes-related foot guidance emphasizing standard definitions, prevention, ulcer classification, infection assessment, and vascular status as determinants of care needs [10,11,12,13,14,15].

### 4.6. Strengths and Limitations

Strengths include a large presentation-level dataset, prespecified derivation of exposure and outcome flags, explicit separation of defining exposure components from baseline comparisons, sensitivity analysis of an expanded profile, and a new exploratory component-category analysis addressing composite heterogeneity. The revised manuscript also reports absolute differences and model discrimination to reduce overinterpretation of statistically significant odds ratios.

Several limitations may affect the findings. First, the analysis is presentation-level and lacks verified unique patient linkage; repeated presentations by the same pilgrim could not be identified or clustered, which may reduce independence of observations and affect precision. Second, documentation bias is possible because chronic conditions and diagnoses depend on recorded fields; diabetes, neuropathy, vascular disease, or prior ulcer history may have been under-documented. Third, the primary outcome is not validated as confirmed surgery and may include surgical assessment, procedural management, or referral-related coding. Fourth, important confounders such as diabetes duration, glycemic control, peripheral arterial disease, wound depth, infection severity, ulcer classification, microbiology, offloading adequacy, baseline functional status, and time-to-care were unavailable, increasing the likelihood of residual confounding and limiting interpretation as independent clinical risk estimation. Fifth, post-Hajj outcomes such as healing, readmission, amputation, infection progression, or completed follow-up were not captured. Sixth, pain score and duration-of-stay variables may include default or documentation-artefact values; they were therefore interpreted descriptively and used cautiously in sensitivity modelling. Finally, rule-based text derivation may misclassify exposure or outcome flags, particularly when clinical documentation is abbreviated or heterogeneous. These limitations mean that the observed associations should be interpreted as care-pathway signals within a presentation dataset rather than causal effects, validated surgery outcomes, or individual-level prognostic estimates.

## 5. Conclusions

Among Hajj pilgrims presenting with foot and ankle conditions, core high-risk foot flags were common and were associated with higher surgery-coded care-intensity documentation and hospital referral. The absolute differences were moderate, the composite was heterogeneous, and model discrimination was limited. The association appeared stronger among ulcer/wound/deep-infection presentations than among chronic-risk-only presentations. These findings support cautious use of high-risk foot flags as operational prompts for assessment, wound-care readiness, and referral pathway planning during Hajj, while emphasizing that the profile is not a validated predictive model, independent clinical risk estimator, or confirmed surgery-risk tool.

## Figures and Tables

**Table 1:** Demographic, pilgrimage, and presentation characteristics by core high-risk profile.

Characteristic	Total (*N* = 3957)	High-Risk (*n* = 1793)	Non-High-Risk (*n* = 2164)	*p*-Value
Age, years, mean (SD)	56.4 (14.1)	57.6 (14.1)	55.4 (14.1)	<0.001
Male sex	1937 (49.0%)	883 (49.2%)	1054 (48.7%)	0.759
BMI, kg/m^2^, mean (SD)	26.8 (4.1)	26.8 (3.9)	26.8 (4.3)	0.789
Previous Hajj/Umrah pilgrimages				<0.001
First time	3017 (76.2%)	1420 (79.2%)	1597 (73.8%)	
1–2	823 (20.8%)	348 (19.4%)	475 (22.0%)	
>2	117 (3.0%)	25 (1.4%)	92 (4.3%)	
Duration of stay in Saudi Arabia, days, median (IQR)	0 (0–1)	0 (0–0)	0 (0–2)	<0.001
Travelling in group	3787 (95.7%)	1762 (98.3%)	2025 (93.6%)	<0.001
Injury location				0.665
Makkah	3058 (77.3%)	1398 (78.0%)	1660 (76.7%)	
Mina	371 (9.4%)	169 (9.4%)	202 (9.3%)	
Arafat	400 (10.1%)	172 (9.6%)	228 (10.5%)	
Muzdalifah	128 (3.2%)	54 (3.0%)	74 (3.4%)	
Pain score, median (IQR)	0 (0–5)	0 (0–5)	0 (0–5)	0.006
Swelling observed	1185 (29.9%)	537 (29.9%)	648 (29.9%)	1.000
Bruising observed	967 (24.4%)	441 (24.6%)	526 (24.3%)	0.862
Deformity observed	701 (17.7%)	276 (15.4%)	425 (19.6%)	<0.001
Footwear problem (worn out/improper/improvised)	2406 (60.8%)	1075 (60.0%)	1331 (61.5%)	0.336

Note: *p*-values are descriptive comparisons. High-risk defining components are intentionally excluded from this table. Duration of stay refers to the recorded duration of stay in Saudi Arabia in days; a value of 0 indicates same-day/less-than-one-day documentation rather than absence of Hajj participation. Pain score was analyzed as recorded; a value of 0 may represent documented absence of pain or a default/undocumented entry in the source record, and pain-score findings were therefore interpreted descriptively.

**Table 2:** Components of the core high-risk foot profile.

Component	n	% of All Presentations	% of High-Risk Profile	Note
Pre-existing diabetes	1040	26.3%	58.0%	Not mutually exclusive
Pre-existing neuropathy	353	8.9%	19.7%	Not mutually exclusive
Diabetic foot ulcer diagnosis	381	9.6%	21.2%	Not mutually exclusive
Foot ulcer diagnosis, unspecified	264	6.7%	14.7%	Not mutually exclusive
Complications of open wound	237	6.0%	13.2%	Not mutually exclusive
Osteomyelitis diagnosis	56	1.4%	3.1%	Not mutually exclusive
Any core high-risk foot profile	1793	45.3%	100.0%	Primary exposure

Note: Components are not mutually exclusive. No *p*-values are shown because these variables define the exposure group.

**Table 3:** Care pathways and secondary outcomes by core high-risk profile.

Outcome/Care Pathway	Total	High-Risk	Non-High-Risk	Absolute Difference	Crude OR	95% CI	*p*-Value	Note
Surgery-coded care-intensity indicator	440 (11.1%)	239 (13.3%)	201 (9.3%)	4.0 pp	1.50	1.23–1.83	<0.001	Primary outcome; treatment field contains surgery
Referral to hospital	431 (10.9%)	219 (12.2%)	212 (9.8%)	2.4 pp	1.28	1.05–1.57	0.017	Treatment field contains referral
Clinical/care-intensity proxy	1750 (44.2%)	828 (46.2%)	922 (42.6%)	3.6 pp	1.16	1.02–1.31	0.026	Confirmed fracture OR deformity OR casting OR referral OR care-intensity indicator
Follow-up planned	939 (23.7%)	410 (22.9%)	529 (24.4%)	−1.5 pp	0.92	0.79–1.06	0.261	Descriptive secondary outcome
Confirmed fracture	82 (2.1%)	41 (2.3%)	41 (1.9%)	0.4 pp	1.21	0.78–1.88	0.453	Radiographic result = fracture
X-ray taken	897 (22.7%)	396 (22.1%)	501 (23.2%)	−1.1 pp	0.94	0.81–1.09	0.448	Care process indicator
Prevented Hajj completion	14 (0.4%)	1 (0.1%)	13 (0.6%)	−0.5 pp	0.09	0.01–0.71	0.009	Rare outcome; interpret cautiously

Note: Crude ORs compare high-risk versus non-high-risk presentation records. pp = percentage points.

**Table 4:** Association between core high-risk profile and surgery-coded care-intensity indicator.

Model	Comparison	OR	95% CI	*p*-Value	n	C-Statistic
Crude	High-risk vs. non-high-risk	1.50	1.23–1.83	<0.001	3957	0.55
Primary adjusted	High-risk vs. non-high-risk	1.47	1.20–1.79	<0.001	3957	0.62
Extended clinical sensitivity	High-risk vs. non-high-risk	1.47	1.20–1.80	<0.001	3957	0.64

Note: Primary adjusted model = high-risk profile + age + sex + BMI + Makkah vs. non-Makkah site. Extended clinical sensitivity model additionally includes pain, footwear problem, swelling, bruising, deformity, and confirmed fracture. C-statistics are reported to describe discrimination only; the models were not developed or validated as prediction tools.

## Data Availability

The data presented in this study are available on request from the corresponding author. Row-level data are not publicly available due to ethical, institutional, and privacy restrictions.

## Supplementary Materials

The following supporting information can be downloaded at: https://www.mdpi.com/article/10.3390/healthcare14121782/s1, Table S1: Sensitivity analysis using expanded high-risk foot definition; Table S2: Derived-variable dictionary; Table S3: Exploratory component and risk-category analysis; Table S4: Site-specific primary outcome counts and site-handling sensitivity models; Table S5: Text/category flag derivation and quality-control notes.
