# Supplementary material for: Core High-Risk Foot Profiles and Surgery-Coded Care-Intensity Indicators Among Hajj Pilgrims Presenting with Foot and Ankle Conditions: A Presentation-Level Analysis
Source: Healthcare (Basel). 2026 Jun 20;14(12):1782. doi: 10.3390/healthcare14121782 (PMC13300411; doi:10.3390/healthcare14121782)
Supplement: Supplementary file 1 [file healthcare-14-01782-s001.zip › healthcare-4379488-supplementary.pdf]

Supplementary Materials

Core High-Risk Foot Profiles and Surgery-Coded Care-Intensity Indicators Among Hajj Pilgrims Presenting With Foot and Ankle Conditions: A Presentation-Level Analysis

This supplementary file contains the expanded high-risk definition sensitivity analysis, derived-variable dictionary, exploratory component/risk-category analysis, site-specific primary outcome counts, site-handling sensitivity models, and text/category flag derivation notes added in response to reviewer comments.

Table S1. Sensitivity analysis using expanded high-risk foot definition.

Definition	Operational definition	n / N (%)	Advanced intervention events	Crude OR	95% CI	p-value	Interpretation
Core high-risk profile	Diabetes OR neuropathy OR diabetic foot ulcer, foot ulcer, open-wound complication, or osteomyelitis	1,793/3,957 (45.3%)	239/1,793 vs. 201/2,164	1.50	1.23-1.83	<0.001	Primary definition
Expanded high-risk profile	Core definition OR injury type = Infection OR pressure ulcer diagnosis	2,330/3,957 (58.9%)	271/2,330 vs. 169/1,627	1.14	0.93-1.39	0.241	Sensitivity only; broader concept weakened the association

Table S2. Derived-variable dictionary.

Derived variable	Operational definition	Primary use
Core high-risk foot profile	Presence of diabetes OR neuropathy OR diabetic foot ulcer OR foot ulcer OR complications of open wound OR osteomyelitis.	Primary exposure
Expanded high-risk foot profile	Core definition OR infection-type injury OR pressure ulcer diagnosis.	Sensitivity analysis only
Surgery-coded care-intensity indicator	Treatment field contains Surgery.	Primary outcome / care-intensity marker
Referral to hospital	Treatment field contains Referral.	Secondary outcome
Clinical/care-intensity proxy	Confirmed fracture OR observed deformity OR casting OR referral OR surgery-coded advanced therapeutic intervention.	Supportive secondary outcome
Planned follow-up	Treatment field contains Follow up / Follow-up.	Descriptive secondary outcome
Confirmed fracture	Fracture confirmed by available clinical/radiographic coding.	Descriptive outcome
Makkah vs. non-Makkah site	Presentation/injury location grouped as Makkah versus all other Hajj sites.	Primary adjusted model covariate

Table S3a. Exploratory component-specific event counts for the surgery-coded care-intensity indicator.

Component	n	Surgery-coded care-intensity events
Pre-existing diabetes	1040	76 (7.3%)
Pre-existing neuropathy	353	50 (14.2%)
Diabetic foot ulcer diagnosis	381	0 (0.0%)
Foot ulcer diagnosis, unspecified	264	82 (31.1%)
Complications of open wound	237	61 (25.7%)
Osteomyelitis diagnosis	56	19 (33.9%)

Note: Components are not mutually exclusive. These counts are exploratory and were not used to redefine the primary exposure.

Table S3b. Exploratory category analysis showing component heterogeneity within the core high-risk foot profile.

Exploratory category	n	Surgery-coded care-intensity events	Crude OR vs non-high-risk	95% CI	p-value
Non-high-risk	2164	201 (9.3%)	Reference	-	-
Chronic risk only	855	77 (9.0%)	0.97	0.73-1.27	0.834
Ulcer/wound/deep infection	938	162 (17.3%)	2.04	1.63-2.55	<0.001

Note: Chronic risk only = diabetes and/or neuropathy without diabetic foot ulcer, foot ulcer, open-wound complication, or osteomyelitis. Ulcer/wound/deep infection = diabetic foot ulcer, foot ulcer, complications of open wound, and/or osteomyelitis. ORs are crude and compare each category with the non-high-risk group.

Table S4a. Site-specific primary outcome event counts.

Site	Total primary outcome events	High-risk events	Non-high-risk events	Interpretation note
Makkah	401/3058 (13.1%)	219/1398 (15.7%)	182/1660 (11.0%)	Largest site; primary model used Makkah vs. non-Makkah
Mina	20/371 (5.4%)	10/169 (5.9%)	10/202 (5.0%)	Sparse events
Arafat	0/400 (0.0%)	0/172 (0.0%)	0/228 (0.0%)	Zero primary outcome events; raw-site models susceptible to separation
Muzdalifah	19/128 (14.8%)	10/54 (18.5%)	9/74 (12.2%)	Sparse denominator

Note: The primary outcome is the surgery-coded care-intensity indicator. Percentages are within site/profile denominators.

Table S4b. Sensitivity analyses evaluating site-handling decisions.

Sensitivity model	Site handling	n	Adjusted OR for high-risk flag	95% CI	p-value	Interpretation
No-site adjustment	Age + sex + BMI; site excluded	3957	1.47	1.21-1.80	<0.001	Consistent with primary model
Primary adjusted model	Age + sex + BMI + Makkah vs. non-Makkah	3957	1.47	1.20-1.79	<0.001	Primary model
Raw-site sensitivity excluding Arafat	Age + sex + BMI + Makkah/Mina/Muzdalifah categorical site	3557	1.47	1.20-1.80	<0.001	Consistent after excluding the zero-event Arafat stratum

Note: Sensitivity models were intended to examine robustness of the high-risk flag association to site handling. They were not used to estimate definitive site effects.

Table S5. Text/category flag derivation and quality-control notes.

Derived flag	Source field(s)	Rule-based derivation	Quality-control note
Core high-risk foot profile flag	Pre-existing conditions and diagnosis fields	Positive if any component flag for diabetes, neuropathy, diabetic foot ulcer, foot ulcer, open-wound complication, or osteomyelitis was positive.	Broad operational flag; not a weighted or validated severity score.
Diabetes flag	Pre-existing conditions	Positive when diabetes was documented in the pre-existing-condition field.	Blank fields were treated as not documented, which may underestimate diabetes.
Neuropathy flag	Pre-existing conditions	Positive when neuropathy was documented in the pre-existing-condition field.	Dependent on documentation quality.
Ulcer/wound/deep-infection category	Diagnosis fields	Positive when diabetic foot ulcer, foot ulcer, open-wound complication, or osteomyelitis was documented.	Used only for exploratory heterogeneity analysis.
Surgery-coded care-intensity indicator	Treatment field	Positive when the treatment field contained Surgery.	Not validated as operating-room surgery; may include procedural care, surgical assessment, or referral-related coding.
Referral to hospital	Treatment field	Positive when referral to hospital was documented in the treatment field.	Secondary care-pathway indicator.
Presentation site	Location field	Standardized to Makkah, Mina, Arafat, or Muzdalifah; primary model used Makkah vs. non-Makkah.	Arafat had zero primary outcome events; site-specific counts and sensitivity models are reported.
Pain score	Pain-score field	Analyzed as recorded.	Zero may reflect documented no pain or default/undocumented entry; interpreted descriptively.

Note: Derivations were rule-based and linked to source fields. A formal independent clinical adjudication sample was not available; this limitation is acknowledged in the manuscript.
